# FOXP3 expression in tumor cells and tumor-infiltrating lymphocytes is associated with breast cancer prognosis

**DOI:** 10.3892/mco.2013.107

**Published:** 2013-04-26

**Authors:** MIKI TAKENAKA, NAOKO SEKI, UHI TOH, SATOSHI HATTORI, AKIHIKO KAWAHARA, TOMOHIKO YAMAGUCHI, KEIKO KOURA, RYUJI TAKAHASHI, HIROKO OTSUKA, HIROKI TAKAHASHI, NOBUTAKA IWAKUMA, SHINO NAKAGAWA, TERUHIKO FUJII, TETSURO SASADA, RIN YAMAGUCHI, HIROHISA YANO, KAZUO SHIROUZU, MASAYOSHI KAGE

**Affiliations:** 1 Departments of Pathology, School of Medicine, Kurume University, Kurume, Fukuoka 830-0011, Japan;; 2 Surgery, School of Medicine, Kurume University, Kurume, Fukuoka 830-0011, Japan;; 3 Research Center for Innovative Cancer Therapy, School of Medicine, Kurume University, Kurume, Fukuoka 830-0011, Japan;; 4 Biostatistical Center, Kurume University, Kurume, Fukuoka 830-0011, Japan;; 5 Department of Diagnostic Pathology, Kurume University Hospital, Kurume, Fukuoka 830-0011, Japan;; 6 Multidisciplinary Treatment Center, Kurume University Hospital, Kurume, Fukuoka 830-0011, Japan;; 7 Department of Immunology, School of Medicine, Kurume University, Kurume, Fukuoka 830-0011, Japan

**Keywords:** breast cancer, clinical prognosis, forkhead box protein 3, regulatory T-cells, tumor-infiltrating lymphocytes

## Abstract

The forkhead box protein 3 (FOXP3) transcription factor is highly expressed in tumor cells as well as in regulatory T cells (Tregs). It plays a tumor-enhancing role in Tregs and suppresses carcinogenesis as a potent repressor of several oncogenes. The clinical prognostic value of FOXP3 expression has not yet been elucidated. In this study, immunohistochemistry was used to investigate the prognostic significance of FOXP3 expression in tumor cells and tumor-infiltrating lymphocytes (TILs) in breast cancer patients. Of the 100 tumor specimens obtained from primary invasive breast carcinoma, 63 and 57% were evaluated as FOXP3^+^ tumor cells and as being highly infiltrated by FOXP3^+^ lymphocytes, respectively. Although FOXP3 expression in tumor cells was of no prognostic significance, FOXP3^+^ lymphocytes were significantly associated with poor overall survival (OS) (n=98, log-rank test P=0.008). FOXP3 exhibited a heterogeneous subcellular localization in tumor cells (cytoplasm, 31%; nucleus, 26%; both, 6%) and, although cytoplasmic FOXP3 was associated with poor OS (P= 0.058), nuclear FOXP3 demonstrated a significant association with improved OS (P=0.016). Furthermore, when patients were grouped according to their expression of tumor cytoplasmic FOXP3 and lymphocyte FOXP3, there were notable differences in the Kaplan-Meier curves for OS (P<0.001), with a high infiltration of FOXP3^+^ lymphocytes accompanied by a cytoplasmic FOXP3^+^ tumor being the most detrimental phenotype. These findings indicated that FOXP3 expression in lymphocytes as well as in tumor cells may be a prognostic marker for breast cancer. FOXP3 in tumor cells may have distinct biological activities and prognostic values according to its localization, which may help establish appropriate cancer treatments.

## Introduction

Forkhead box protein 3 (FOXP3) is a member of the forkhead/winged-helix family of transcription factors involved in the regulation of the development and function of the immune system (1,2). The human *FOXP3* gene is located on the short arm of the X chromosome and consists of 11 translated exons encoding a protein of 431 amino acids (3). It contains a proline-rich N-terminal repressor domain that suppresses the expression of target genes, a zinc-finger and a leucine-zipper motif that allow FOXP3 homo- or heterodimerization and a conserved DNA-binding forkhead domain (FKH) with two sites that target the nuclear localization of FOXP3 at its C- and N-termini (3,4).

FOXP3 plays a crucial role in the generation of immunosuppressive CD4^+^CD25^+^ regulatory T cells (Tregs), which induce immune tolerance to antigens (2,5). Loss of FOXP3 function leads to Treg deficiency, resulting in lethal autoaggressive lymphoproliferation, whereas FOXP3 overexpression leads to severe immunodeficiency (2,5). FOXP3-expressing Tregs are reportedly abundant in the tumor infiltrates and peripheral blood of cancer patients (4,6,7). They are also involved in the immune evasion mechanisms promoted by cancer. Studies on several types of cancer suggested that high levels of Treg infiltration of the tumor bed are associated with poor clinical outcome (4,8–12).

FOXP3 protein expression was initially considered to be restricted to the lymphocyte lineage. However, its expression has been demonstrated in various types of non-hematopoietic cells, including human tumor cells (13–16). Although previous studies suggested that *FOXP3* is an X-linked tumor suppressor gene in the breast (16) and prostate gland (17), its biological function and importance in tumor cells have not been elucidated. Previous studies suggested that tumor-expressed FOXP3 may be useful as a clinical prognostic marker. For example, wild-type FOXP3 from normal cells, unlike mutated FOXP3 from cancer cells, bound to and transcriptionally repressed human epidermal growth factor receptor (HER) 2 and S-phase kinase-associated protein (SKP) 2 (16,18) or c-Myc (17) oncogenes involved in mammary or prostate carcinogenesis, respectively. Furthermore, FOXP3 overexpression in human cancer cell lines was shown to repress tumor growth (16,18,19) and FOXP3 was reported to be a key determinant of tumor suppression in p53-dependent responses to DNA-damaging chemotherapeutic agents (20).

In a different context of tumor-expressed FOXP3, Hinz *et al* previously reported that FOXP3 expression in a pancreatic cancer cell line inhibited the proliferation of anti-CD3/anti-CD28-stimulated T cells without impeding their activation (14). This finding suggested that tumor-infiltrating Tregs influence antitumor immunity (4,8–12) and that tumor cells may modulate T-cell function and trigger a mechanism of immune evasion through FOXP3.

FOXP3 is constitutively expressed in the nucleus of human Tregs (4,8–12). By contrast, previous immunohistochemical studies indicated that FOXP3 cytoplasmic expression was more abundant, compared to nuclear expression, in several types of cancer, including breast carcinoma (14,21–25). Conflicting prognostic values for tumor-expressed FOXP3 were reported in immunohistochemical studies of breast cancer, in which FOXP3 was associated with poor (23), as well as with favorable prognosis (21). Therefore, the prognostic value of FOXP3 expression in breast cancer remains controversial. The present study immunohistochemically investigated the prognostic relevance of FOXP3 expression in tumor cells and tumor-infiltrating lymphocytes (TILs) in breast cancer patients.

## Materials and methods

### Materials

A total of 100 adult females with primary invasive breast carcinoma who underwent breast surgery at our institution (Kurume University Hospital, Kurume, Japan) between 1995 and 2005 and who had not received neoadjuvant chemotherapy, were enrolled in the present study. Hematoxylin and eosin (H&E)-stained histological sections from each patient were analyzed for biological parameters and histological grading was performed using the Nottingham-combined histological grade [Scarff-Bloom-Richardson (SBR) grading system] (26). Table I provides the clinicopathological characteristics of the patients. The study was approved by our institutional review board and written informed consent was obtained from all enrolled patients. All data were anonymized.

### Immunohistochemical staining

The estrogen receptor (ER) and progesterone receptor (PgR) status were analyzed immunohistochemically on formalin-fixed, paraffin-embedded tumor sections, using ER (clone SP1) and PgR (clone 1E2) antibodies at a dilution of 1:100 and the iVIEW system (Ventana Medical Systems, Tucson, AZ, USA). Labeling was detected using the Ventana BenchMark XT automat (Ventana Medical Systems). The arrays were counterstained with hematoxylin. The HercepTest scoring method with the 4B5 antibody (Ventana Medical Systems) was used to determine the HER2 status, with a score of 3+ or 2+ with fluorescent *in situ* hybridization (FISH) amplification, as determinants of HER2-overexpressing tumors.

FOXP3 expression was immunohistochemically analyzed using rat anti-human FOXP3 monoclonal antibody clone ab22510 (Abcam, Cambridge, UK). Paraffin-embedded tissue samples were cut into 4-μm sections and examined on a coated glass slide. Intrinsic peroxidase activity was blocked by treatment with peroxidase-blocking reagent (DakoCytomation, Glostrup, Denmark) for 5 min. The specimens were boiled in a microwave for 30 min in 1 mmol/l EDTA (pH 9.0) target retrieval solution (DakoCytomation), to recover the antigens. After washing in Tris-buffered saline (TBS; DakoCytomation) for 10 min, the FOXP3 antibody was diluted 1:600 and applied to the specimens. Histological specimens were incubated at 4°C overnight, washed in TBS for 15 min and incubated with labeled polymer-horseradish peroxidase (HRP) secondary antibody (ChemMate Envision kit; DakoCytomation) for 30 min at room temperature. After washing in TBS for 10 min, the slides were visualized using 3,3′-diaminobenzidine.

**Figure 1:**
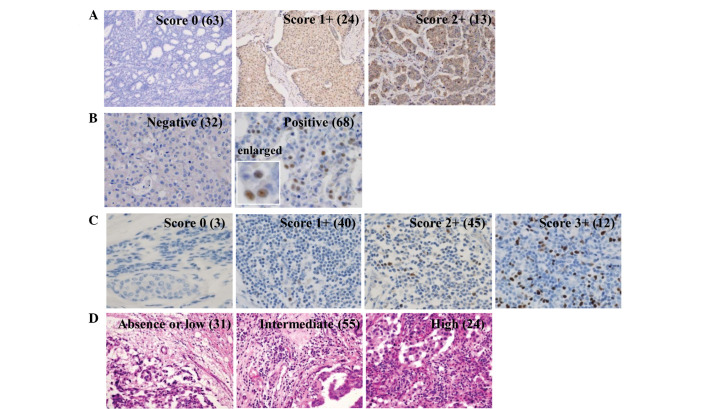
Immunohistochemical forkhead box protein 3 (FOXP3) staining in breast cancer. Representative images of FOXP3 expression in (A) the cytoplasm (magnification, ×100) and (B) the nucleus (magnification, ×400) of tumor cells and (C) the lymphocytic infiltrate (magnification, ×400). (D) Hematoxylin and eosin (H&E) staining intensity of tumor-infiltrating lymphocytes (TILs) (magnification, ×200). The number of specimens in each graded group is indicated in parenthesis. A score of 1+/2+ in (A) or 2+/3+ in (C) was defined as positive for tumor-cytoplasmic FOXP3 or lymphocyte FOXP3 (i.e., high infiltration of FOXP3^+^ lymphocytes).

FOXP3 expression was evaluated independently by two authors (M.T and M.K), who were blinded to the clinico-pathological data. Discrepancies were reviewed jointly and a consensus was reached. The staining intensity of FOXP3-positivity (FOXP3^+^) within the tumor-cell cytoplasm was scored as weak (1+) or strong (2+) (Fig. 1A). The number of FOXP3+ cells present within tumor-cell nuclei were counted manually in 10 high-power fields (HPFs; magnification, ×400) (Fig. 1B). The extent of FOXP3^+^ TILs was scored as follows: no positive cells, 0; 1–25% positive cells, 1+; 26–50% positive cells, 2+; and 51–100% positive cells, 3+ (Fig. 1C). TIL H&E staining intensity was determined in intratumor nodules and in the surrounding stroma and was defined as absent, low, intermediate or strong (Fig. 1D).

### Statistical analysis

Overall survival (OS) was defined as the time period between the time of surgery and the time of death from any cause. Patients who were alive at the last contact attempt were regarded as censored cases at this time point. Relapse-free survival (RFS) was defined as the time period from the time of surgery until progressive disease was confirmed by magnetic resonance imaging (MRI) or computed tomography (CT), or until death from any cause. Patients without progressive disease were regarded as censored cases at the date of their last CT or MRI examination.

For tumor-cell cytoplasm FOXP3 expression, a score of 0 was defined as negative and scores of 1+ or 2+ as positive. For lymphocyte FOXP3 expression, scores of 0 and 1+ were defined as negative (absent or low infiltration) and scores of 2+ and 3+ as positive (high infiltration). These definitions accounted for the median score and minimized the difference between the number of patients classified as negative and those classified as positive. For tumor nuclear FOXP3 expression, ≥30% was defined as positive and <30% as negative from a statistical viewpoint. In the Cox regression model with a binary explanatory variable representing positive or negative with various cut-off points, we selected the value maximizing the profile partial likelihood, i.e., we selected the cut-off value that provided the best fit to the OS data using various classifications. Associations between FOXP3 expression in tumor cells and lymphocytes and between FOXP3 expression and clinicopathological factors were examined with the Fisher’s exact test. Survival functions for OS and RFS were estimated with the Kaplan-Meier method and compared with the log-rank test. Cox regression analysis was performed to examine whether FOXP3 expression was associated with OS or RFS following adjustment for possible confounding factors. Clinicopathological characteristics significantly associated with FOXP3 expression were included in the Cox regression for adjustment.

Statistical analyses were conducted with SAS version 9.2 (SAS Institute Inc., Cary, NC, USA) and R version 2.9.0. P<0.05 was considered to indicate a statistically significant difference.

## Results

### FOXP3 expression in breast cancer specimens

Of the 100 tumor specimens immunostained for FOXP3, 63 (63%) and 57 (57%) were evaluated as positive for expression in tumor cells and TILs, respectively. FOXP3 was expressed in the nucleus of lymphocytes, representing Treg infiltration, whereas a heterogeneous subcellular localization of FOXP3 was observed in tumor cells (i.e., the cytoplasm and/or nucleus; Table II). Most FOXP3 staining in tumor cells was localized to the cytoplasm [31 (31%)] or the nucleus [26 (26%)] and 6 specimens (6%) exhibited FOXP3 expression in the cytoplasm and nucleus (P=0.014). By contrast, no significant positive correlation was observed between FOXP3 expression in tumor cells and high infiltration of FOXP3^+^ lymphocytes. FOXP3 expression in TILs was significantly correlated with an absence of nuclear FOXP3 expression in tumor cells (P=0.002).

Table III shows the frequency of prognostic clinicopathological characteristics according to the presence or absence of FOXP3 immunostaining. Cytoplasmic FOXP3 expression in tumor cells was significantly associated with larger tumor size (P=0.035) and presence of metastatic lymph nodes (P=0.015), whereas nuclear FOXP3 expression in tumor cells was significantly associated with ER positivity (P=0.003). A high infiltration by FOXP3^+^ lymphocytes was significantly associated with tumor grade III (P<0.001), HER2 positivity (P=0.03), ER negativity (P<0.001) and a triple-negative phenotype (ER^−^/PgR^−^/HER2^−^) (P=0.003).

### Prognostic significance of FOXP3 expression in breast cancer

Prognostic analysis was performed using the 98 patients whose clinical outcome was monitored. Univariate analysis of clinicopathological characteristics indicated that high tumor grade (III) and ER negativity were significantly associated (P<0.05) with mortality (OS), whereas no significant prognostic value for OS was observed when the other factors were assessed (Table IVA). FOXP3 expression in tumor cells (cytoplasm and/or nucleus) showed no prognostic significance [hazard ratio (HR): 1.19; 95% confidence interval (CI): 0.45–3.13; P=0.722]. However, FOXP3^+^ lymphocytes were significantly associated with worse OS (HR: 5.87; 95% CI: 1.34–25.69; P=0.008). Notably, the prognostic values of tumor-cell FOXP3 expression were determined according to FOXP3 localization; nuclear FOXP3 expression was significantly associated with improved OS (HR: 0.13; 95% CI: 0.02–0.95; P=0.016). Inversely, borderline significance was observed between the association of tumor-cell cytoplasmic FOXP3 expression and poor OS (HR: 2.47; 95% CI: 0.94–6.50; P=0.058).

**Figure 2:**
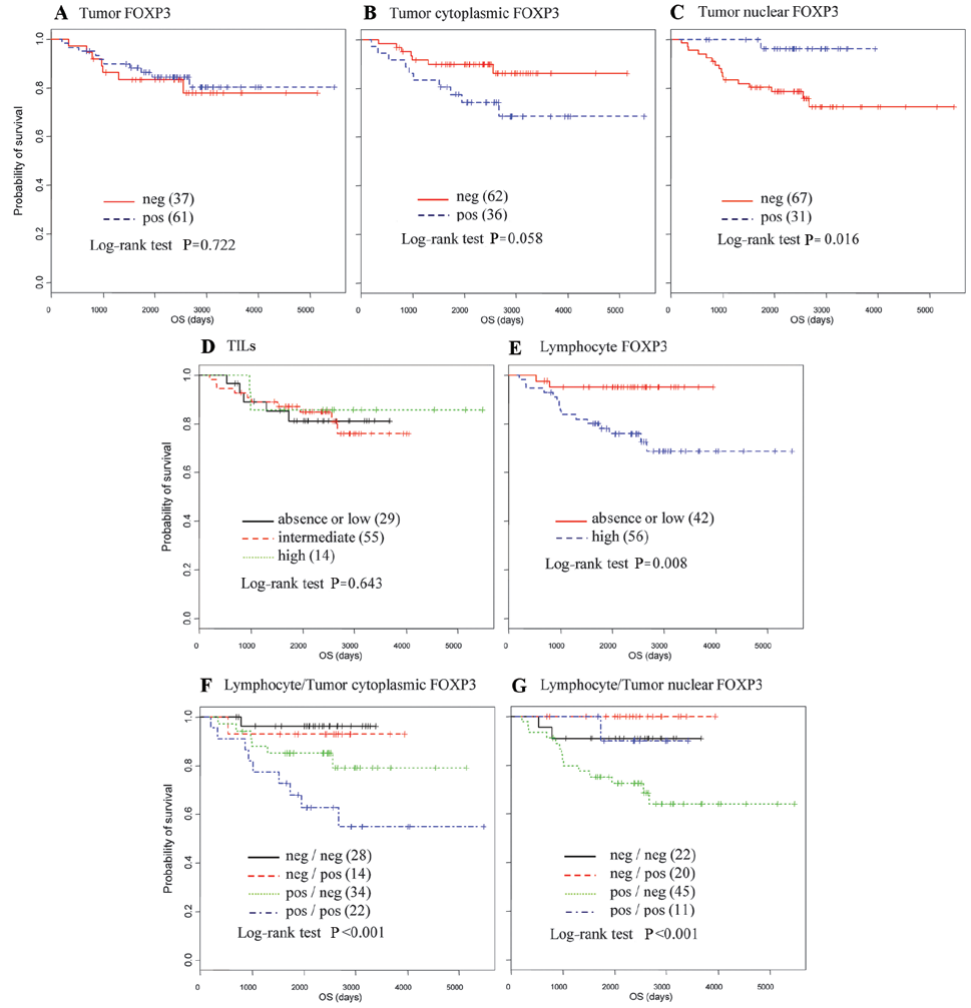
Kaplan-Meier curves for overall survival (OS) associated with forkhead box protein 3 (FOXP3) expression in breast cancer. Kaplan-Meier curves in two groups divided into (A) FOXP3-positive (+/−, −/+ and +/+, cytoplasmic/nuclear expression) and -negative (−/−), (B) cytoplasmic FOXP3-positive (+/− and +/+) and -negative (−/+ and −/−) and (C) nuclear FOXP3-positive (−/+ and +/+) and -negative (+/− and −/−) expression in tumor cells. Intensities of (D) tumor-infiltrating lymphocytes (TILs) and (E) FOXP3^+^ lymphocytes were stratified as follows: absent-low, intermediate and high infiltration in (D); negative (absent-low) and positive (high infiltration of FOXP3^+^ lymphocytes) in (E). Kaplan-Meier curves in the four groups according to infiltration of (F) FOXP3^+^ lymphocytes and cytoplasmic or (G) nuclear FOXP3 in tumor cells. Number of specimens in each group is shown in parenthesis. P-values were calculated using the log-rank test. neg, negative; pos, positive.

Kaplan-Meier curves confirmed that FOXP3 expression localized in the cytoplasm or nucleus of tumor cells was associated with worse (log-rank test, P=0.058) or improved (log-rank test, P=0.016) OS, respectively (Fig. 2A–C). By contrast, the intensity of lymphocyte infiltration of the tumor site was not associated with OS (Fig. 2D); however, a larger number of FOXP3^+^ lymphocytes conferred a significantly worse OS (log-rank test P=0.008; Fig. 2E), suggesting a crucial role for FOXP3^+^ Tregs in tumor progression. FOXP3 expression in tumor cells and lymphocytes exhibited the same tendency for prognostic value based on the risk of relapse-free survival (RFS) (data not shown). When survival was analyzed in the four subgroups classified according to FOXP3 localization in tumor cells (−/−, +/−, −/+ and +/+, cytoplasm/nucleus FOXP3 expression), positive patients with either cytoplasmic or nuclear FOXP3 staining were found to have a similar, worse, or improved outcome compared to the negative (−/−) group (data not shown).

Multivariate analysis of the covariates with P<0.05 in Table III, indicated that FOXP3 expression in TILs (HR: 4.96; 95% CI: 1.07–23.06; P=0.041) was an independent prognostic factor for OS, unlike FOXP3 localization in tumor cells (cytoplasm, HR: 2.68; 95% CI: 0.90–7.97; P=0.077; and nucleus, HR: 0.15; 95% CI: 0.02–1.16; P=0.070) (Table IVB). Patients exhibited significant differences in Kaplan-Meier curves in OS (log-rank test, P<0.001; Fig. 2F), demonstrating a more detrimental effect on the prognosis of patients exhibiting cytoplasmic FOXP3^+^ tumor cells as well as a high infiltration of FOXP3^+^ lymphocytes, compared to the effect of either factor alone. This combined phenotype was identified as a significant, independent risk factor for OS (HR: 4.22; 95% CI: 1.39–12.82; P=0.011) by multivariate analysis of the possible confounding factors in Table IV (data not shown). By contrast, nuclear FOXP3 expression in tumor cells appeared to attenuate the negative effect of FOXP3^+^ lymphocyte accumulation on OS (Fig. 2G).

## Discussion

FOXP3^+^ Tregs are immunosuppressive, therefore, their abundance in tumor infiltrates is associated with an unfavorable clinical outcome. Several previous studies reported that increased infiltration of FOXP3^+^ lymphocytes in the tumor microenvironment was associated with poor prognosis in cancer patients (4,8–12). However, several studies demonstrated conflicting results (27–29) and it should be noted that not all FOXP3^+^ TILs are Tregs, since T-cell receptor (TCR) activation of conventional T cells may induce the transient expression of FOXP3 without suppressive properties (4). Although the association between accumulated FOXP3^+^ TILs and clinical prognosis can be beneficial or detrimental, depending on the type of malignancy under investigation, the present findings clearly indicate that a high density of FOXP3^+^ lymphocytes in tumor tissue is a strong, independent prognostic marker associated with mortality, a finding consistent with those of previous studies on breast cancer (8–12).

The present study has demonstrated that FOXP3 localization in breast cancer was crucial to predicting clinical outcome. Zuo *et al* previously demonstrated that ∼80% of normal breast samples expressed FOXP3 in the epithelial cell nuclei, whereas only 20% of cancer tissues expressed nuclear FOXP3 (i.e., mostly the HER2^−^ or ER^+^ phenotype) (16), which is consistent with our findings (Tables II and III). Furthermore, predominant cytoplasmic FOXP3 staining of tumor cells was demonstrated in several types of cancer (14,15,21,23–25), although its relevance has not been clarified. Two previously conducted representative immunohistological studies demonstrated that FOXP3 staining of breast cancer specimens was localized either completely (21) or predominantly in the tumor-cell cytoplasm, with only a few specimens exhibiting nuclear staining (23). In one of these reports (21), cytoplasmic FOXP3 expression was associated with improved OS and RFS in HER2-overexpressing patients; however, this contrasted our findings and those of Merlo *et al* (23). This discrepancy may be partly due to the similarities between the study populations included in our study and those included in the study by Merlo *et al* (the patients had not undergone neoadjuvant chemotherapy) and the ratios of HER2 overexpression-harbored patients (our study, 23%; two clinical trials by Merlo *et al*, 15 and 22%). Our study demonstrated more nucleus-specific FOXP^+^ expression, which may be associated with an ER^+^/HER2^−^ phenotype and improved clinical outcome.

The underlying mechanism(s) by which the expression of tumor FOXP3 affects prognosis require further investigation. Zuo *et al* reported a high proportion of somatic mutations or deletions of the *FOXP3* gene in human breast cancer cells, which may include the nuclear localization signals surrounding the FKH domain of FOXP3 (16). A previous study by Wang *et al* demonstrated that three out of the four FOXP3 mutants obtained from human pancreatic carcinomas exhibited disrupted translocation into the nuclei and were instead localized in the cytoplasm (17). Localization in the cytoplasm may therefore be a functional deficiency or modulation of the tumor suppressor *FOXP3* gene. This may account for our finding that cytoplasmic, unlike nuclear, FOXP3 expression in tumor cells was associated with detrimental clinical outcome.

Accumulating evidence indicates that FOXP3 coordinates with multiple transcriptional regulators and its localization may depend on its molecular partners (30). Viewing FOXP3 as a multifaceted factor of cancer biology may provide another explanation for its bifacial prognostic value. Further investigations are required to determine whether the heterogeneous subcellular localization of tumor FOXP3 is functionally relevant to the clinical prognosis.

A previous study suggested that tumor-expressed FOXP3 triggers a mechanism for the immune evasion of tumor cells (14). High infiltration of FOXP3^+^ lymphocytes accompanied by a cytoplasmic FOXP3^+^ tumor was the most detrimental phenotype, although the FOXP3^+^ lymphocytes and the tumor were not significantly correlated in this study. FOXP3 may propagate crosstalk between tumor cells and their immunological microenvironment, e.g., involving signal transducer and activator of transcription 3 (STAT3) (31), leading to tumor-induced immunosuppression, including the induction of Tregs. By contrast, nuclear FOXP3 expression in tumor cells, which was associated with improved OS in this study, was significantly enhanced in patients with absent or low infiltration of FOXP3^+^ lymphocytes. Hinz *et al* also demonstrated that the downregulation of FOXP3 led to the upregulation of the pro-inflammatory cytokines interleukin (IL)-6 and IL-8 in human pancreatic carcinoma cell lines (14). Since these cytokines are known to influence the progression of breast cancer (32,33), the regulation of cytokine synthesis by nuclear FOXP3 may affect the interaction between tumor cells and their microenvironment and, subsequently, clinical prognosis.

Our data suggested that FOXP3 expression in tumor cells and TILs may be an effective prognostic marker in breast cancer patients and that FOXP3 localization in tumor cells is an important determinant of prognosis. FOXP3 may provide distinct biological activities and prognostic values according to its localization. However, multivariate analysis demonstrated that FOXP3 expression in TILs, unlike that in tumor cells, was an independent prognostic factor for OS (Table IVB). However, cytoplasmic or nuclear FOXP3^+^ tumor cells may also be associated with OS, as the correlation was at ∼5% significance level, following adjustment for possible confounding factors. The relatively small patient sample may have limited the statistical power of the present study and future investigations including a larger sample size are required to confirm the results. Our findings may facilitate the selection of appropriate patient treatments and assist in the designing of FOXP3-targeted therapeutic strategies for breast cancer.

## Figures and Tables

**Table I:** Clinicopathological characteristics of breast cancer patients.

Characteristic	n (%)
Total no. of patients	100
Age (years)	
≤50	29 (29)
>50	71 (71)
Tumor size (cm)	
≤2.0	59 (59)
>2.0	41 (41)
Axillary nodal status	
Positive	45 (45)
Negative	47 (47)
Resection not performed	8 (8)
Tumor grade	
I and II	77 (77)
III	23 (23)
HER2	
Positive	23 (23)
Negative	77 (77)
ER	
Positive	56 (56)
Negative	44 (44)
Triple-negative^a^	21 (21)

a ER^−^/PgR^−^/HER2^−^ phenotype. HER2, human epidermal growth factor receptor 2; ER, estrogen receptor.

**Table II:** Localization of FOXP3 expression.

	Tumor-cell cytoplasm			Tumor-cell nucleus		
	
	FOXP3^+^	FOXP3^−^		FOXP3^+^	FOXP3^−^	
	
	Patient no. (%)	Patient no. (%)	P-value	Patient no. (%)	Patient no. (%)	P-value
Total patient no.	37	63		32	68	
Tumor-cell cytoplasm						
FOXP3^+^				6 (18.8)	31 (45.6)	0.014
FOXP3^−^				26 (81.3)	37 (54.4)	
Tumor-cell nucleus						
FOXP3^+^	6 (16.2)	26 (41.3)	0.014			
FOXP3^−^	31 (83.8)	37 (58.7)				
Lymphocytes^a^						
FOXP3^+^	23 (62.2)	34 (54.0)	0.531	11 (34.4)	46 (67.6)	0.002
FOXP3^−^	14 (37.8)	29 (46.0)		21 (65.6)	22 (32.4)	

Evaluated by Fisher’s exact test.

a FOXP^+^, high infiltrate; FOXP3^−^, absent-low infiltrate of FOXP3-expressing lymphocytes. FOXP3, forkhead box protein 3.

**Table III:** Frequency of patient clinicopathological characteristics according to FOXP3 expression.

	Tumor-cell cytoplasm			Tumor-cell nucleus			Lymphocytes^a^		
		
	FOXP3^+^	FOXP3^−^		FOXP3^+^	FOXP3^−^		FOXP3^+^	FOXP3^−^	
		
Characteristic	n/total (%)	n/total (%)	P-value	n/total (%)	n/total (%)	P-value	n/total (%)	n/total (%)	P-value
Age, years (>50 years)	24/37 (64.9)	45/63 (71.4)	0.510	22/32 (68.8)	47/68 (69.1)	1.000	38/57 (66.7)	31/43 (72.1)	0.664
Tumor size (>2 cm)	21/37 (56.8)	21/63 (33.3)	0.035	11/32 (34.4)	31/68 (45.6)	0.386	25/57 (43.9)	17/43 (39.5)	0.688
LN metastasis	21/31 (67.7)	24/61 (39.3)	0.015	13/32 (40.6)	32/60 (53.3)	0.279	28/51 (54.9)	17/41 (41.5)	0.216
Tumor grade III	8/37 (21.6)	15/63 (23.8)	1.000	4/32 (12.5)	19/68 (27.9)	0.126	21/57 (36.8)	2/43 (4.70)	<0.001
HER2-positive	10/37 (27.0)	13/63 (20.6)	0.472	4/32 (12.5)	19/68 (27.9)	0.126	18/57 (31.6)	5/43 (11.6)	0.030
ER-positive	18/37 (48.6)	38/63 (60.3)	0.300	25/32 (78.1)	31/68 (45.6)	0.003	21/57 (36.8)	35/43 (81.4)	<0.001
Triple-negative^b^	8/37 (21.6)	13/63 (20.6)	1.000	4/32 (12.5)	17/68 (25.0)	0.193	18/57 (31.6)	3/43 (7.0)	0.003

Evaluated by Fisher’s exact test.

a FOXP^+^, high infiltrate; FOXP3^−^, absent-low infiltrate of FOXP3-expressing lymphocytes,

b ER^−^/PgR^−^/HER2^−^ phenotype. LN, lymph node; FOXP3, forkhead box protein 3.

**Table IV:** Univariate and multivariate analyses (Cox regression) for overall survival.

A, Univariate analysis.			
Variable	HR	95% CI	P-value

Age (>50 years)	0.61	0.23–1.61	0.318
Tumor size (>2 cm)	2.06	0.78–5.41	0.135
LN metastasis	1.74	0.63–4.78	0.279
Tumor grade III	3.05	0.94–9.36	0.040
ER-positive	0.38	0.14–1.04	0.050
HER2-positive	2.35	0.89–6.17	0.075
Triple-negative^a^	1.39	0.45–4.27	0.565
Tumor FOXP3^+, b^	1.19	0.45–3.13	0.722
Cytoplasmic	2.47	0.94–6.50	0.058
Nuclear	0.13	0.02–0.95	0.016
Lymphocyte FOXP3^+,c^	5.87	1.34–25.69	0.008
Intensity of TILs			
High vs. absent-low	0.71	0.14–3.68	0.685
Intermediate vs. absent-low	1.01	0.35–2.96	0.984

B, Multivariate analysis.			

Variable	HR	95% CI	P-value

Tumor cytoplasmic FOXP3^+^	2.68	0.90–7.97	0.077
Tumor size (>2 cm)	1.66	0.57–4.84	0.355
LN metastasis	1.20	0.41–3.47	0.739
Tumor nuclear FOXP3^+^	0.15	0.02–1.16	0.070
ER-positive	0.51	0.19–1.39	0.185
Lymphocyte FOXP3^+,c^	4.96	1.07–23.06	0.041
Tumor grade III	0.88	0.27–2.92	0.836
ER-positive	0.70	0.17–2.91	0.621
HER2-positive	1.42	0.38–5.33	0.606

a ER^−^/PgR^−^/HER2^−^ phenotype;

b cytoplasmic and/or nuclear FOXP3^+^;

c high infiltrate of FOXP^+^ lymphocytes. HR, hazard ratio; CI, confidence interval; LN, lymph node; ER, estrogen receptor; HER2, human epidermal growth factor receptor 2; FOXP3, forkhead box protein 3; TIL, tumor-infiltrating lymphocyte.

## References

[1] Coffer PJ, Burgering BM (2004). Forkhead-box transcription factors and their role in the immune system. Nat Rev Immunol.

[2] Hori S, Nomura T, Sakaguchi S (2003). Control of regulatory T cell development by the transcription factor Foxp3. Science.

[3] Lopes JE, Torgerson TR, Schubert LA (2006). Analysis of FOXP3 reveals multiple domains required for its function as a transcriptional repressor. J Immunol.

[4] Martin F, Ladoire S, Mignot G, Apetoh L, Ghiringhelli F (2010). Human FOXP3 and cancer. Oncogene.

[5] Sakaguchi S, Ono M, Setoguchi R (2006). Foxp3^+^CD25^+^CD4^+^natural regulatory T cells in dominant self-tolerance and autoimmune disease. Immunol Rev.

[6] Liyanage UK, Moore TT, Joo HG (2002). Prevalence of regulatory T cells is increased in peripheral blood and tumor microenvironment of patients with pancreas or breast adenocarcinoma. J Immunol.

[7] Woo EY, Chu CS, Goletz TJ (2001). Regulatory CD4(+)CD25(+) T cells in tumors from patients with early-stage non-small cell lung cancer and late-stage ovarian cancer. Cancer Res.

[8] Bates GJ, Fox SB, Han C (2006). Quantification of regulatory T cells enables the identification of high-risk breast cancer patients and those at risk of late relapse. J Clin Oncol.

[9] Bohling SD, Allison KH (2008). Immunosuppressive regulatory T cells are associated with aggressive breast cancer phenotypes: a potential therapeutic target. Mod Pathol.

[10] Curiel TJ, Coukos G, Zou L (2004). Specific recruitment of regulatory T cells in ovarian carcinoma fosters immune privilege and predicts reduced survival. Nat Med.

[11] Gobert M, Treilleux I, Bendriss-Vermare N (2009). Regulatory T cells recruited through CCL22/CCR4 are selectively activated in lymphoid infiltrates surrounding primary breast tumors and lead to an adverse clinical outcome. Cancer Res.

[12] Ohara M, Yamaguchi Y, Matsuura K, Murakami S, Arihiro K, Okada M (2009). Possible involvement of regulatory T cells in tumor onset and progression in primary breast cancer. Cancer Immunol Immunother.

[13] Ebert LM, Tan BS, Browning J (2008). The regulatory T cell-associated transcription factor FoxP3 is expressed by tumor cells. Cancer Res.

[14] Hinz S, Pagerols-Raluy L, Oberg HH (2007). Foxp3 expression in pancreatic carcinoma cells as a novel mechanism of immune evasion in cancer. Cancer Res.

[15] Karanikas V, Speletas M, Zamanakou M (2008). Foxp3 expression in human cancer cells. J Transl Med.

[16] Zuo T, Wang L, Morrison C (2007). FOXP3 is an X-linked breast cancer suppressor gene and an important repressor of the HER-2/ErbB2 oncogene. Cell.

[17] Wang L, Liu R, Li W (2009). Somatic single hits inactivate the X-linked tumor suppressor FOXP3 in the prostate. Cancer Cell.

[18] Zuo T, Liu R, Zhang H (2007). FOXP3 is a novel transcriptional repressor for the breast cancer oncogene SKP2. J Clin Invest.

[19] Zhang HY, Sun H (2010). Up-regulation of Foxp3 inhibits cell proliferation, migration and invasion in epithelial ovarian cancer. Cancer Lett.

[20] Jung DJ, Jin DH, Hong SW (2010). Foxp3 expression in p53-dependent DNA damage responses. J Biol Chem.

[21] Ladoire S, Arnould L, Mignot G (2011). Presence of Foxp3 expression in tumor cells predicts better survival in HER2-overexpressing breast cancer patients treated with neoadjuvant chemotherapy. Breast Cancer Res Treat.

[22] Liang YJ, Liu HC, Su YX (2011). Foxp3 expressed by tongue squamous cell carcinoma cells correlates with clinicopathologic features and overall survival in tongue squamous cell carcinoma patients. Oral Oncol.

[23] Merlo A, Casalini P, Carcangiu ML (2009). FOXP3 expression and overall survival in breast cancer. J Clin Oncol.

[24] Tao H, Mimura Y, Aoe K (2012). Prognostic potential of FOXP3 expression in non-small cell lung cancer cells combined with tumor-infiltrating regulatory T cells. Lung Cancer.

[25] Winerdal ME, Marits P, Winerdal M (2011). FOXP3 and survival in urinary bladder cancer. BJU Int.

[26] Elston CW, Ellis IO (1991). Pathological prognostic factors in breast cancer. I. The value of histological grade in breast cancer: experience from a large study with long-term follow-up. Histopathology.

[27] Badoual C, Hans S, Rodriguez J (2006). Prognostic value of tumor-infiltrating CD4^+^T-cell subpopulations in head and neck cancers. Clin Cancer Res.

[28] Carreras J, Lopez-Guillermo A, Fox BC (2006). High numbers of tumor-infiltrating FOXP3-positive regulatory T cells are associated with improved overall survival in follicular lymphoma. Blood.

[29] Salama P, Phillips M, Grieu F (2009). Tumor-infiltrating FOXP3^+^ T regulatory cells show strong prognostic significance in colorectal cancer. J Clin Oncol.

[30] Zhou Z, Song X, Li B, Greene MI (2008). FOXP3 and its partners: structural and biochemical insights into the regulation of FOXP3 activity. Immunol Res.

[31] Yu H, Kortylewski M, Pardoll D (2007). Crosstalk between cancer and immune cells: role of STAT3 in the tumour microenvironment. Nat Rev Immunol.

[32] Knupfer H, Preiss R (2007). Significance of interleukin-6 (IL-6) in breast cancer (review). Breast Cancer Res Treat.

[33] Nicolini A, Carpi A, Rossi G (2006). Cytokines in breast cancer. Cytokine Growth Factor Rev.

